# Tibial intramedullary nailing in the lateral decubitus position: Technical notes and preliminary clinical outcomes

**DOI:** 10.1097/MD.0000000000021234

**Published:** 2020-07-10

**Authors:** Lei Xu, Wanbo Zhu, Kai Xie, Lei Liu, Xianzuo Zhang, Jiazhao Yang, Xujin Wang, Shiyuan Fang

**Affiliations:** a Department of Orthopaedics, The First Affiliated Hospital of University of Science and Technology of China; b Department of Orthopaedics, Affiliated Anhui Provincial Hospital of Anhui Medical University, Hefei, People's Republic of China.

**Keywords:** infrapatellar approach, intramedullary nailing, lateral decubitus position, tibial shaft fracture

## Abstract

**Background:**

Traditional intramedullary nailing (IMN) for tibial shaft fractures through an infrapatellar approach is typically performed in the supine position and requires a specially designed operative table and an experienced assistant throughout the surgery. We attempted to perform IMN for tibial fractures in the lateral decubitus position to make the process easier both for surgeons and radiographers.

**Methods:**

A total of 36 tibial IMN procedures were performed with the patient in the lateral position from May 1, 2014 to April 30, 2016. The technical feasibility and early results were evaluated.

**Results:**

The mean time to complete the nailing procedure during surgery was 78.4 ± 1.1 min. The mean intraoperative time for fluoroscopy was 36.7 ± 1.1 min. No radiographic angular malalignment or bone non-union was reported. No surgical site infections or other surgery-related complications occurred in our series.

**Conclusion:**

Tibial IMN through an infrapatellar approach in the lateral decubitus position may be a valuable alternative as it simplifies the procedure for both surgeons and radiographers. This technique is highly effective for surgical operation and fluoroscopy compared to traditional supine position. This technique also seems to provide satisfactory clinical and radiographic outcomes in our preliminary clinical outcomes.

## 1 Introduction

Intramedullary nailing (IMN) is one of the most common methods for closed tibial shaft fractures.^[1]^ Biomechanical advantages of IMN and minimally invasive incisions reduce the incidence of non-union and allow for early functional rehabilitation.^[2]^

Traditional tibial IMN through an infrapatellar approach is performed with the patient in the supine position. Use of traction or other off-traction variations of the supine position is associated with satisfactory results. However, operating in the supine position calls for some requirements in surgical equipment, space, and experienced assistance. For example, the knee of the injured leg has to be flexed over a radiolucent bolster on an operative table with a radiolucent edge. The C-arm needs to have an adequate diameter to rotate from above the flexed knee to the bottom of the operative table. A sizeable operative space is necessary because the guide wire placement and nail insertion are performed above the shoulder height of the surgeon. An experienced assistant must crouch and hold the leg for stable reduction until the nail is passed beyond the fracture.^[3]^

In this study, we share a new and easily replicable surgical method for tibial IMN through an infrapatellar approach in the lateral decubitus position for fractures of the middle and distal tibial shaft. As a supplementary technique for traditional IMN, tibial IMN in the lateral decubitus position may simplify the surgical process, particularly when space is limited and effective traction is difficult to achieve.

## 2 Patients and methods

**Figure 1:**
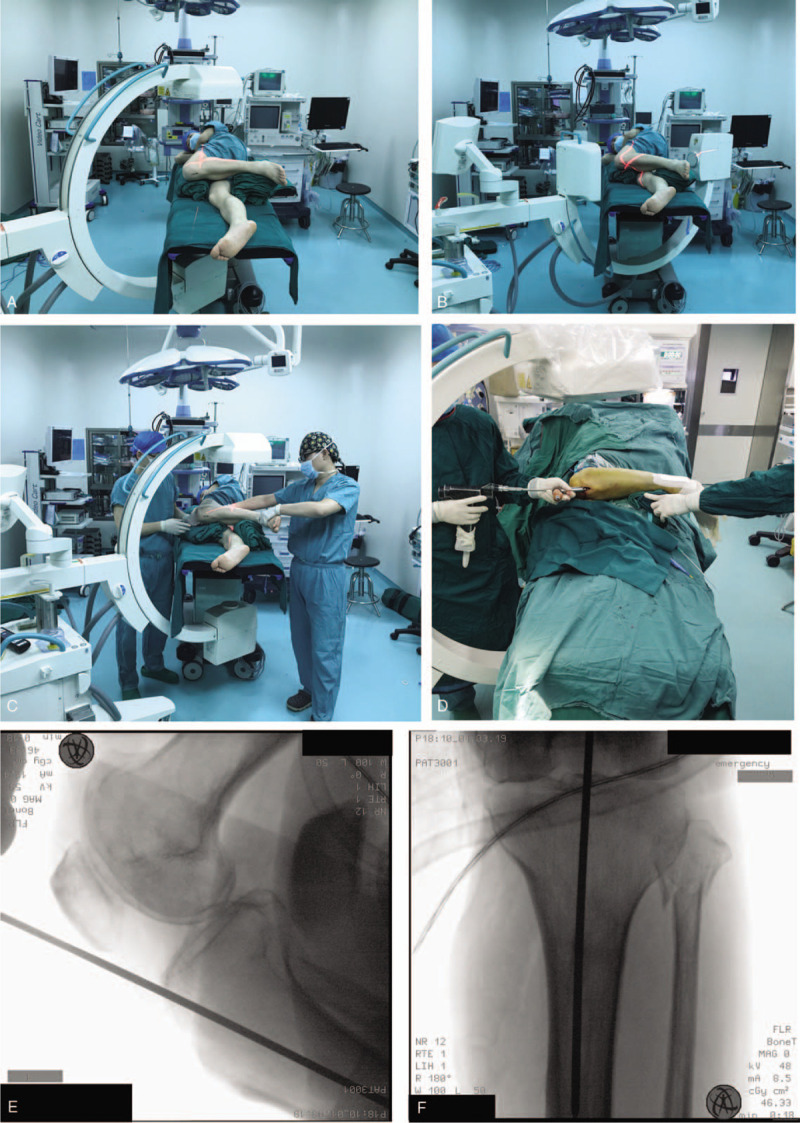
Lateral decubitus position for proximal tibial IMN in initial steps. (A and B) Schematic of AP and LAT tibial fluoroscopy for percutaneous entry point in the lateral decubitus position. (C) Schematic of bone reduction maintaining for guide wire placement. (D) Guide wire placement in the lateral position. (E and F) AP and LAT image of approach point.

This was a retrospective study of IMN for tibial fractures conducted in our hospital. Patients included in this report consented to have their clinical data collected and used for publication. The inclusion criteria were patients with a closed tibial shaft and distal extra-articular fractures (AO 42 and AO 43-A) or simple intra-articular fractures (AO 43-B) that could be successfully treated with screw fixation. The exclusion criteria were patients with ipsilateral femoral fracture or hip fracture who could not change the position of the injured leg during distal locking. A total of 36 tibial IMN procedures were performed with the patients in the lateral position between May 1, 2014 and April 30, 2016. All patients were operated on by Dr Xu and his group. Electronic radiological pictures were captured using the C-arm (ZIEHM Imaging, Nuremberg, Germany). Each patient was placed in the lateral decubitus position with the help of positioners on a non-specific surgical table. The injured leg was placed with the knee flexed, on top of the uninjured straight leg (shown in Fig. 1A and B), similar to the position used for injections into the gluteus maximus, to achieve flaccidity of the gluteus maximus. Radiolucent dressings or bolsters were used to support and maintain the horizontal position of the medial border of the injured leg. The C-arm was advanced and remained in a suitable position in front of the patient throughout the operation. Anteroposterior and lateral tibial imaging were achieved by swinging the C-arm without pedestal movement. This study was approved by the ethics committee of the First Affiliated Hospital of USTC in 2014. The number of ethical approval was AHSLYY20-P-035. As for this research, an optout of the informed consent, the information disclosure, and a negative opportunity are guaranteed in the Ethical approval.

**Figure 2:**
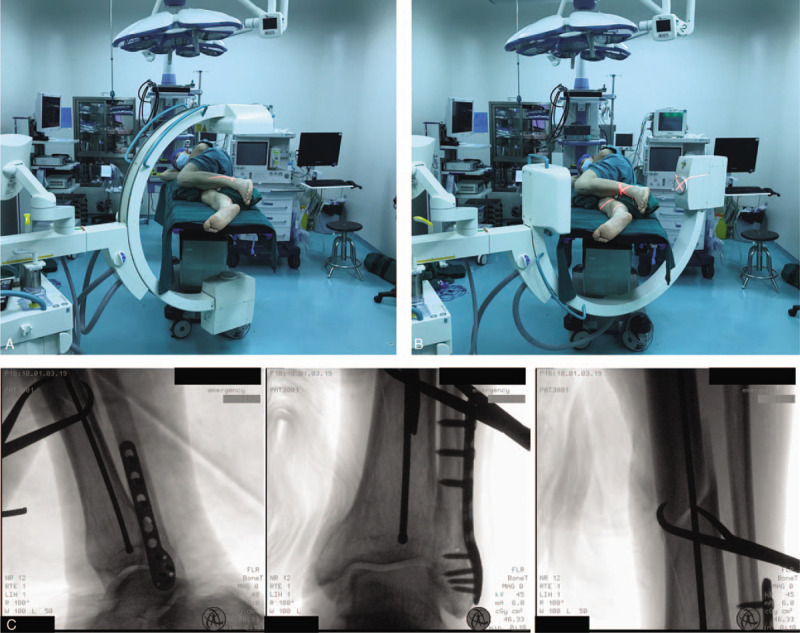
Lateral decubitus position for distal tibial IMN in initial steps. (A and B) Schematic of AP and LAT tibial fluoroscopy for bone reduction and distal tibia wire placement in the lateral decubitus position. (C) AP and LAT image of wire placement passed beyond the fracture in distal tibia.

**Figure 3:**
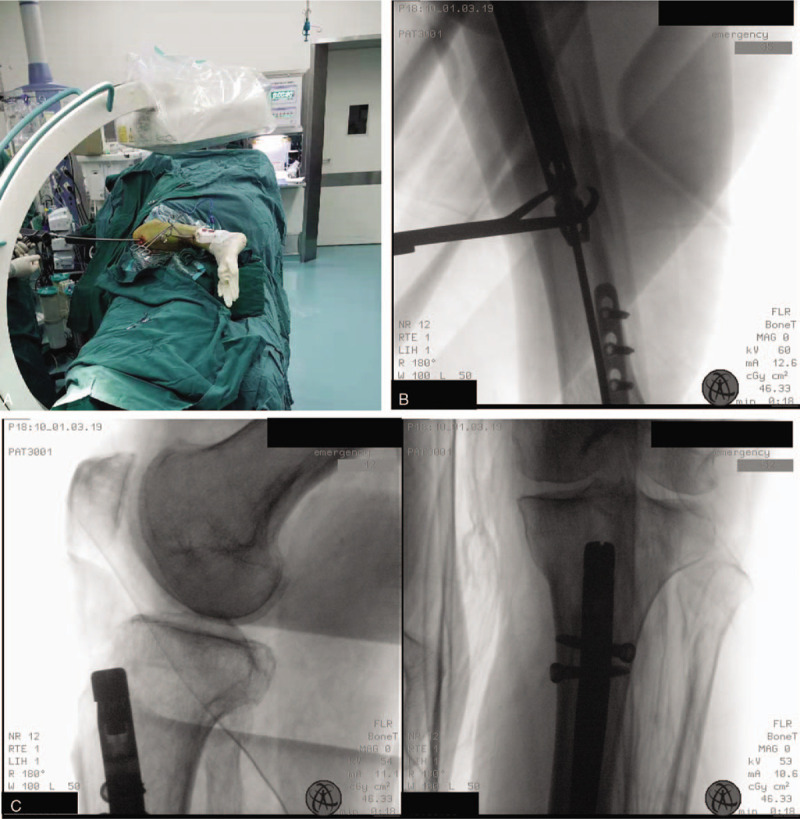
Lateral decubitus position for tibial IMN in insertion and locking steps. (A) Reaming and nail insertion in the lateral decubitus position. (B) Nail passed beyond the fracture. (C) AP and LAT image of proximal nail locking.

**Figure 4:**
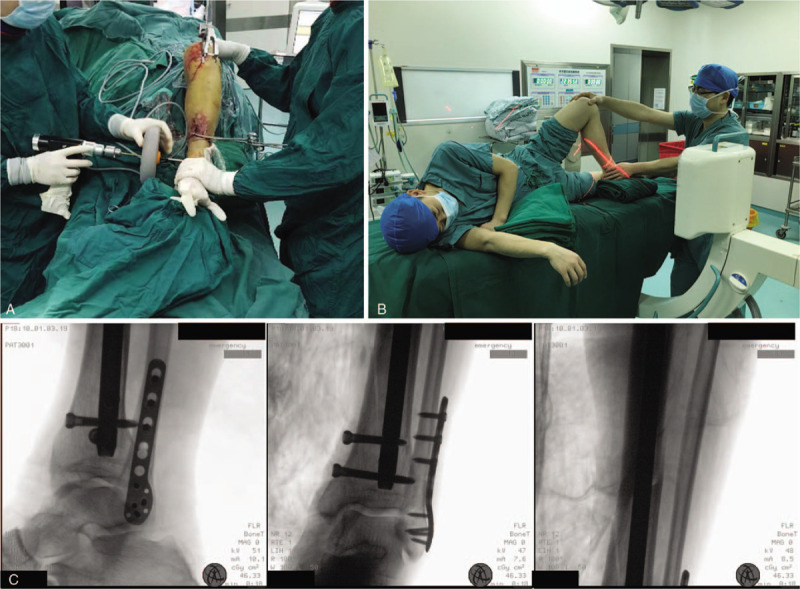
Lateral decubitus position for tibial IMN in distal locking step. (A and B) Distal locking and its schematic in the lateral decubitus position. (C) AP and LAT image of distal nail locking and fracture reduction after nail pass.

**Figure 5:**
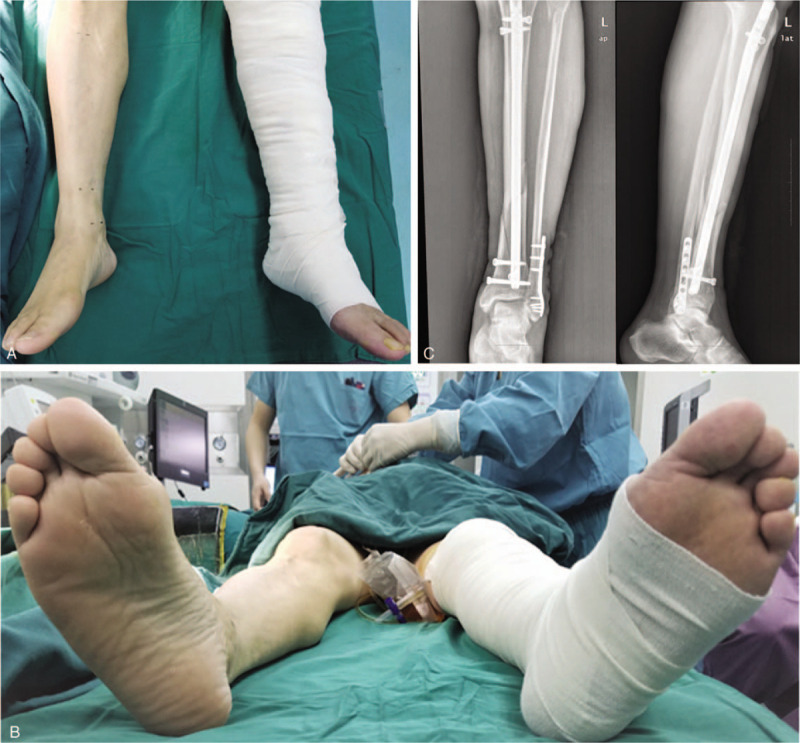
Postoperative assessment of the lateral decubitus position for tibial IMN. (A and B) Limb length and rotation after the operation. (C) postoperative radiology soon after the operation.

All intraoperative procedures and fluoroscopy were conducted with the patient in the lateral position, and the operative procedure was basically consistent with traditional tibial IMN through an infrapatellar approach. Percutaneous entry point approach and guide wire placement are shown in Figure 1C. For displacement and angulation in the coronal direction, auxiliary dressings and positioners were employed to stabilize the force in this direction. Anteroposterior shifting and rotation were adjusted and temporarily fixed by clamps. Necessary traction was maintained by an assistant until the guide wire passed beyond the fracture (Fig. 1D). Distal tibial images provided a reference for the wire placement (shown in Fig. 2). After the guide wire was passed beyond the fracture, reaming and insertion of the nail were also performed in this position (shown in Fig. 3). Nail locking in the lateral position was similar to that in the supine position (shown in Fig. 4). An X-ray-free distal aiming device is an alternative device for surgeons and patients. Auxiliary positioning frames and locking under fluoroscopic guidance are also optional in this position. Assessments of limb length and rotation were performed soon after the operation (shown in Fig. 5). Acceptable radiographic alignment was defined as angulation <5° in any plane, and any detectable malrotation was considered unacceptable.^[4]^

## 3 Results

All patients were followed for at least 1 year. The mean time to complete the nailing procedure during surgery was 78.4 ± 1.1 min, and the mean intraoperative time for fluoroscopy was 36.7 ± 1.1. No open reductions of the tibial shaft fractures were required. Seven patients underwent fibular fracture fixation before IMN. No radiographic angular malalignment >5° was seen in the coronal or sagittal planes in any case. Bony union was achieved in all patients. No surgical site infections or other surgery-related complications were seen. Table 1 presents the clinical data of patients included in our study group by fracture type.

**Table 1:**
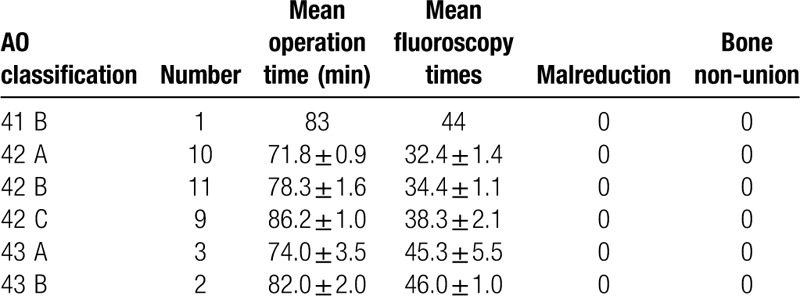
Clinical data.

## 4 Discussion

IMN is considered the treatment of choice for tibial shaft fractures in adults. Improvements to the conventional tibial IMN technique are ongoing, and various techniques have been proposed.^[1,5]^ Suprapatellar and semi-extended nailing techniques were proposed as an alternative to the traditional infrapatellar approach and have achieved satisfactory clinical outcomes.^[6]^ However, there are limitations to a retropatellar approach, including chondral damage to the patella and articular cavity debris.^[7,8]^ A second infrapatellar approach to remove the device may be performed.^[9]^ Conventional tibial IMN through an infrapatellar approach is still widely applied in some areas and has unique advantages. In this study, we attempted to modify this traditional technique by performing tibial IMN through an infrapatellar approach for middle and distal shaft fractures in the lateral decubitus position. As a supplementary technique, this technique may simplify the operative procedure for both surgeons and assistants, particularly when space is limited and effective traction devices are difficult to acquire.

To maintain proper surgical ergonomics and asepsis, the initial steps in the supine position are challenging. Steps such as guide wire placement, reaming, and nail insertion are performed above the shoulder height of most surgeons. A long lever arm makes it difficult for the surgeon to take control of the entry point, reamer, and nail. When performed in the lateral position, the steps mentioned above are performed with the surgeon's arm held below the shoulder height at a desired level. This greatly reduces procedural difficulty for the surgeon. In 2014, Granville-Chapman et al reported a freehand “Figure 4” technique for tibial IMN.^[10]^ Less fatigue, fast and accurate guide wire placement, and swift reamer size changes were noted as advantages associated with this technique during the initial steps of IMN.

Intraoperative fluoroscopy for the tibia is also simplified in the lateral decubitus position. Tibial images can be acquired by rotating the C-arm in only the vertical and horizontal planes to achieve unobstructed images. In the traditional supine position, the radiographer must rotate the C-arm around the tibia for satisfactory positioning and image quality, depending on the different angles of the supportive bolster. In some modified techniques, images are captured by moving the limb. This requires an experienced assistant and can cause movement at the fracture site.

When performed in the traditional supine position, the knee needs to be flexed and positioned vertically on the table and held by an assistant. Angulation and rotation can occur in any direction. When performed in the lateral position, the force in the coronal direction is constant. Angulation and rotation in this direction can be stabilized by using suitable positioners. The assistant does not need to hold the leg and only needs to focus on adjusting for sagittal displacement. Notably, for distal tibial fractures, IMN is often associated with more malalignment than plating.^[11]^ Complex distal fractures might require a combination of fixation IMN and plate support to achieve satisfactory and stable reduction.^[12–14]^ From the lateral decubitus position, it is easier to reduce and fix the fibula.

Tibial IMN in the lateral decubitus position confines the operative procedures to the surgical table with no need to expand the surgical area beyond the table edge. Thus, an unconventional pedestal and radiolucent bed edge are not necessary. A C-arm with a larger diameter is also not required since the procedure is performed within a limited working space, in contrast to the traditional method. Rotation of the C-arm only occurs in the vertical and horizontal planes with the base moved proximally and distally along the table. This simplifies the ergonomic considerations for the radiographer.

The limitations of this modified technique are the same as the conventional supine infrapatellar approach. First, problems of poor reduction of proximal fractures and infrapatellar nerve injury still exist in this position. For some distal metaphyseal fractures with significant angulation, poor longitudinal traction in the lateral position might cause difficulties in maintaining satisfactory limb alignment.^[11]^ Additionally, changing the position of the injured leg during distal locking cannot be performed in patients with ipsilateral femoral fractures or hip fractures. For example, when external rotation of the hip joint is limited, this technique cannot be utilized. Finally, for some fractures, such as extreme shortening and prolonged fractures, auxiliary reduction and traction devices could facilitate the reduction procedure. However, these devices are generally used in patients in the traditional supine position, so the lateral position may not be suitable. Further research is in progress to compare the efficacy and complications of this lateral position technique with the traditional technique through complete follow-up.

## 5 Conclusions

Tibial IMN in the lateral decubitus position simplifies the operative procedure both for surgeons and assistants. This modified technique also improves the shortcomings of reduction and traction in the supine position when specific devices are difficult to acquire.

## Author contributions

XL, ZWB, YJZ, and ZXZ conceived and designed the study. ZWB, YJZ, ZXZ, and LL collected the data. ZWB wrote the manuscript. XL and FSY read, corrected, and approved the final manuscript. All authors read and approved the final manuscript.

**Conceptualization:** Shiyuan Fang.

**Data curation:** Kai Xie.

**Formal analysis:** Lei Liu.

**Investigation:** Lei Liu, Xianzuo Zhang.

**Methodology:** Lei Xu.

**Supervision:** Jiazhao Yang.

**Writing – original draft:** Wan-bo Zhu, Kai Xie, Xianzuo Zhang, Jiazhao Yang.

**Writing – review & editing:** Xianzuo Zhang, Xujin Wang, Shiyuan Fang.
